# Single photon emission from graphene quantum dots at room temperature

**DOI:** 10.1038/s41467-018-05888-w

**Published:** 2018-08-27

**Authors:** Shen Zhao, Julien Lavie, Loïc Rondin, Lucile Orcin-Chaix, Carole Diederichs, Philippe Roussignol, Yannick Chassagneux, Christophe Voisin, Klaus Müllen, Akimitsu Narita, Stéphane Campidelli, Jean-Sébastien Lauret

**Affiliations:** 1 Laboratoire Aimé Cotton, CNRS, Univ. Paris-Sud, ENS Cachan, Université Paris-Saclay, bat 505 campus d’Orsay, 91405 Orsay cedex, France; 2 LICSEN, NIMBE, CEA, CNRS, Université Paris-Saclay, Gif sur Yvette, 91191 France; 3 Laboratoire Pierre Aigrain, Département de physique de l’ENS, École normale supérieure, Université Paris Diderot, Sorbonne Paris Cité, Sorbonne Université, CNRS, PSL University, 75005 Paris, France; 4 Max Planck Institute for Polymer Research, Ackermannweg 10, 55128 Mainz, Germany

## Abstract

Graphene being a zero-gap material, considerable efforts have been made to develop semiconductors whose structure is compatible with its hexagonal lattice. Size reduction is a promising way to achieve this objective. The reduction of both dimensions of graphene leads to graphene quantum dots. Here, we report on a single-emitter study that directly addresses the intrinsic emission properties of graphene quantum dots. In particular, we show that they are efficient and stable single-photon emitters at room temperature and that their emission wavelength can be modified through the functionalization of their edges. Finally, the investigation of the intersystem crossing shows that the short triplet lifetime and the low crossing yield are in agreement with the high brightness of these quantum emitters. These results represent a step-forward in performing chemistry engineering for the design of quantum emitters.

## Abstract (web summary)

The optical properties of nanographenes can be engineered by designing their size, shape, and edges. Here, the authors show that graphene quantum dots are single photon emitters at room temperature, and their emission wavelength can be controlled by edge functionalization.

## Introduction

Graphene plays a central role as an emerging material for nanoelectronics. Nevertheless, graphene is a semimetal, which constitutes a severe limitation for some future applications. Therefore, considerable efforts are being made to develop semiconductor materials whose structure is compatible with the graphene lattice. In this perspective, little pieces of graphene are very promising^1,2^. In particular, their electronic, optical, and spin properties can be in principle controlled by designing their size, shape and edges^3–6^.

To date, graphene quantum dots (GQDs) have been mostly synthesized by top–down approaches, such as the oxidation of carbon fibers or graphene^7,8^. These methods permit to produce easily large quantities of nanoparticles but they also present important drawbacks that make them not suitable to produce emitters with well-defined properties. In particular, they do not allow to control neither the size nor the chemical nature of the edges of the dots. Therefore, their optical properties are dominated by defect states instead of the intrinsic confined states of the nanographene^9^. Fortunately, over the last two decades, bottom–up synthesis have been developed opening the way to a precise control of the GQD structure^2^. Several sizes and shapes of GQDs have already been synthesized^1^. However, these materials often face problems, in particular aggregation, that hinder the possibility to make the link between their structure and their intrinsic properties, especially in solid state or at the single-emitter level^1,10^.

Here, we report on a single-emitter study that directly addresses the intrinsic emission properties of GQDs synthesized by this bottom–up approach. We show that graphene quantum dots are efficient and stable single-photon emitters at room temperature. A control of the emission wavelength (∼100 nm redshift) is obtained through the functionalization of the graphene quantum dot edges. Finally, the intersystem crossing (ISC) has been investigated. The short triplet lifetime and the low ISC yield agree with the high brightness of these new quantum emitters. These results represent a step-forward in performing chemistry engineering for the design of quantum emitters.

## Results and discussion

### Samples

**Fig. 1:**
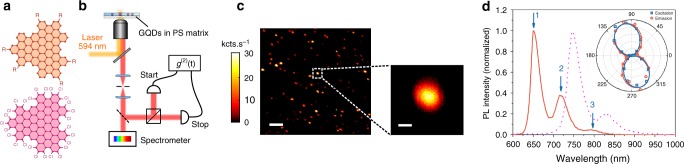
Photoluminescence of single GQDs. **a** Chemical structure of the C_96_ and C_96_Cl GQDs. *R* stands for C_12_H_25_. **b** Scheme of the microphotoluminescence setup. **c** 20 × 20 μm^2^ PL map of the C_96_ GQDs in polystyrene matrix. The color bar represents the number of counts per second on the APD. The scale bar is 3 μm. The zoom shows a diffraction limited spot that can be fitted with a 2D Gaussian function leading to a 1/e^2^ diameter of ∼600 nm. The scale bar is 200 nm. **d** Room temperature PL spectra of a single C_96_ GQD (solid line) and of a single C_96_Cl GQD (dotted line). Polarization diagram in excitation (blue) and emission (red)

The chemical structure of the two type of GQDs studied here is displayed on Fig. 1a. They are both made of 96 sp^2^ carbon atoms arranged in a triangular shape which leads to lateral sizes of ∼2 nm. Six alkyl chains (R = C_12_H_25_) were introduced at the edges of the first GQD to enhance its solubility. The edges of the second GQD have been functionalized with chlorine atoms to modify the optical properties. In the following, we call the two structures C_96_ and C_96_Cl GQDs, respectively. The details of the synthesis have been already reported^3,11^ and are described in the Supplementary Methods section. Briefly, the C_96_ GQD is synthesized in two steps from the 1,3,5-triethynylbenzene and 2,5-diphenyl-3,4-di(4-dodecylphenyl)-cyclopentadien-1-one via Diels-Alder cycloaddition followed by oxidative cyclodehydrogenation in the presence of FeCl_3_ (See Supplementary Fig. 1). The C_96_Cl GQD is synthesized in three steps from the 1,3,5-triethynylbenzene and 2,3,4,5-tetraphenyl-cyclopentadien-1-one via Diels-Alder cycloaddition followed by the oxidation of the dendrimer via the Scholl reaction and the chlorination of the bare C_96_ particle by treatment with ICl in the presence of AlCl_3_ (see Supplementary Fig. 1). The intermediate compounds are fully characterized and the GQDs are characterized by MALDI-TOF spectrometry to confirm the complete dehydrogenation and chlorination (See Supplementary Fig. 2). The GQDs are dispersed in 1,2,4-trichlorobenzene and then mixed with a solution of polystyrene (PS). The mixture is subsequently spin-coated on a coverslip to perform the optical experiments, using the experimental setup described in Fig. 1b. Complementary details about the sample preparation can be found in the Methods section.

### Photoluminescence spectroscopy

Figure 1c shows an example of a photoluminescence (PL) map of C_96_ GQDs embedded in PS matrix for the highest dilution used in this study (see also Supplementary Fig. 10). One can observe bright spots (25 kcounts s^−1^ with 200 nW excitation) with diffraction limited size. The PL spectrum acquired on such a spot is displayed on Fig. 1d. For this particular C_96_ GQD, the spectrum is composed of three lines, noted 1, 2, and 3, centered at 653, 719, and 797 nm, respectively. The full width at half maximum of the main line is of the order of 27 nm (80 meV). Note that the wavelength as well as the relative intensity of the PL lines slightly vary from one GQD to another, which is certainly due to differences in their local environments. Also, averaging over 25 GQDs gives an energy splitting of 170 ± 5 meV between lines 1 and 2, and 165 ± 10 meV between lines 2 and 3. Moreover, as mentioned in the introduction, one of the great potential of GQDs lies in the precise tuning of their electronic properties through the control of their structure. As a first example, the dotted curve in Fig. 1d shows the PL spectrum of a single C_96_Cl GQD whose emission wavelength has been shifted by the chemical functionalization of the edges with chlorine atoms. Here the functionalization leads to an almost 100 nm rigid redshift of the main PL line. This observation is in agreement with theoretical predictions that show a decrease of the optical gap of C_96_Cl in comparison with C_96_ GQD due to the electronegativity of the chlorine atoms^11^. This result illustrates how the intrinsic properties of GQDs can be controlled by the synthesis.

In the following, we focus on C_96_ GQDs containing the C_12_H_25_ alkyl chains. First we investigate the nature of the quantum states at the origin of the three PL lines. The energy splitting between each line is ∼170 meV very close to the C=C stretching vibration mode. Moreover, theoretical works on this family of objects indicate the existence of low energy dark states, the lowest one being called the *α* band in the Clar’s notation^12,13^. The coupling with the vibrations of the lattice can lead to a brightening of these low energy states^14^. Here, we attribute the first PL line to the zero-phonon emission line of the *α* band followed up with vibronic replicas, with a quantum of vibration *ħ*Ω ∼ 170 meV. We also performed photoluminescence excitation (PLE) experiments in solution (See Supplementary Fig. 4). The PLE curves detected on the two highest energy PL lines of GQD superimpose well with the absorption spectrum of the solution. Besides, the PLE highlights the existence of two lines at 580 and 630 nm. The energy splitting between those states is also ∼170 meV revealing the Franck–Condon series of vibronic lines. The intense absorption line at high energy could be tentitatively attributed to the *β* band in the Clar’s notation^12,13^. Finally, the polarization response of a single GQD is shown in the inset of Fig. 1d. Here, the emission polarization profile is recorded on the line 1 at ∼650 nm. It is linearly polarized at a fixed direction certainly related to the geometry of the GQD. Likewise the excitation diagram is also linearly polarized in the same direction than the emission one. Therefore, it can be concluded that absorption and main emission dipoles are parallel. Further investigations, including theoretical modeling, are needed to explain this experimental observation.

### Second-order correlation function

**Fig. 2:**
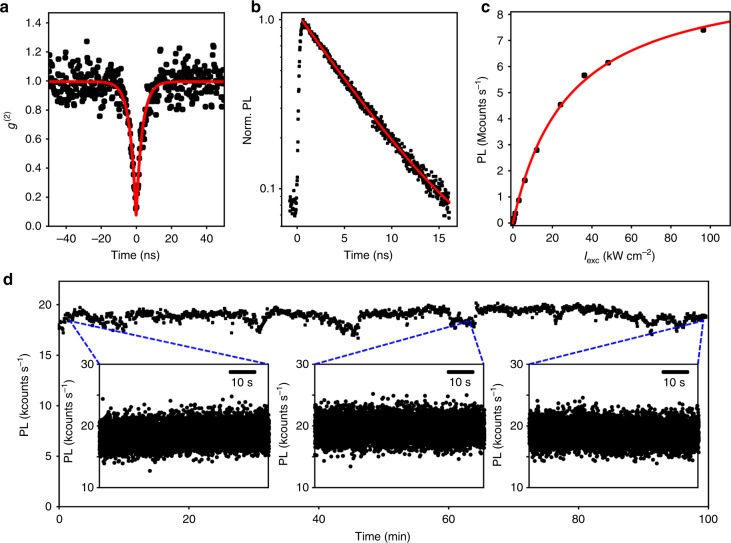
Photophysics of a single GQD. **a** Second-order correlation function *g*^(2)^(*τ*) recorded from a diffraction limited spot such as the one of Fig. 1 (black dots), showing a strong antibunching. A fit (red line) with a function $1 - (1 - b)e^{ - |\tau |/\tau _1}$ yields *g*^(2)^(0) = 0.05 ± 0.05 and a characteristic time of *τ*_1_ ∼ 3.5 ns (FWHM of the IRF of the detector ∼0.9 ns). **b** Time-resolved PL of a single GQD (black dots) detected on the whole spectrum, fitted by a mono-exponential decay (red line) with a time constant *τ* = 5.37 ns. **c** Saturation curve of a single GQD (black dots) as a function of the pump power. A fit by Eq. (1) (red line) leads to a saturation power density of 28 kW cm^−2^ and a saturation intensity *I*_sat_ ∼ 9.7 Mcounts/s. **d** PL time trace of a stable GQD over 100 min with a binning time of 200 ms. Fluctuations are due to setup instabilities. Zooms are shown on shorter timescale with a binning time of 10 ms

In order to identify the number of emitters associated with such diffraction limited spot and spectrum, we measured the second-order correlation function (*g*^(2)^(*τ*)) at room temperature integrating over the entire spectrum. As displayed in Fig. 2a, the strong antibunching observed at zero delay, *g*^(2)^(0) < 0.1, is a proof that a single emitter is detected. This is in strong contrast with the results of such experiments performed on top–down GQDs where no antibunching is observed^9^. In these earlier studies, the absence of antibunching is interpreted as a consequence of the extrinsic nature of the states at the origin of the luminescence, leading to multidefect sites emitting in an uncorrelated manner^9^. We have performed measurements on more than 30 GQDs, all of them leading to *g*^(2)^(0) < 0.1 (see examples on Supplementary Fig. 5). Moreover, these correlation measurements being performed by integrating all the wavelengths on the detector, implies that the spectrum described above actually arises from a single GQDs and not from several objects. Moreover, the weak value observed for the *g*^(2)^(0) is an indication of the good purity of single-photon emission associated with single GQD. This result enforces GQD as an interesting alternative to other single emitters^15^, such as defects in WSe_2_^16–20^, in *h*-BN^21–23^ or in carbon nanotubes^24^.

### Photophysical properties

The fact that we are actually observing single objects allows us to characterize other important properties. First, another figure of merit of a single quantum emitter is its brightness. A saturation curve of a single GQD is displayed on Fig. 2c. The intensity is fitted by

1 $$R = R_{{\mathrm{sat}}}{\mathrm{/}}\left( {1 + \frac{{I_{{\mathrm{sat}}}}}{{I_{{\mathrm{exc}}}}}} \right),$$

with *R*_sat_ the count rate at saturation, *I*_sat_ the incident power density at saturation and *I*_exc_ the incident power density. The fit leads to *R*_sat_ ∼ 9.7 Mcounts s^−1^, and *I*_sat_ = 28 kW cm^−2^. This value of count rate at saturation can be compared to other new quantum emitters such as defects in 2D materials. In this context, L.J. Martínez et al. have compared on the same setup the count rate at saturation of N-V centers and single defects in *h*-BN^22^. We decided to employ the same method in order to quantitatively compare GQDs and single defects in *h*-BN (see Supplementary Note 3 for details). It turns that GQDs are, at least, as bright as the brightest single-photon source found in 2D materials. Therefore, it puts GQDs in the highest values of brightness among other quantum emitters^15,22,23^. Next, we address the question of the photostability of GQDs. In this perspective, GQDs have also good properties. Indeed, photostability up to hours have been observed for an incident power of 200 nW (0.12 kW cm^−2^) and an emission rate of ∼20 kcounts.s^−1^ (see Fig. 2d and Supplementary Note 5). This encouraging result could even be improved by engineering the surrounding matrix, as it has been done for small molecules^25,26^. Moreover, Fig. 2d shows that no blinking is observed on the time trace of the luminescence of this GQD. The histogram of intensity fits well to a normal distribution (see Supplementary Note 4 and Supplementary Fig. 7). This observation is in strong contrast with numerous single emitters that undergo quantum jumps between bright and dark states. Nevertheless, the luminescence of GQDs end up disappearing. Supplementary Fig. 8 shows two different time traces around the time of bleaching. In the first one, the luminescence drops down to zero sharply. On the contrary, the second one goes through an intermediate gray state before ending up to zero. These two behaviors are representative of what we observed on the GQDs. The discrete intensity jumps are also characteristic of single-emitter experiments. In order to get insights in the recombination dynamics, time-resolved photoluminescence (TR-PL) experiments on single GQDs have been performed (see Fig. 2b and Supplementary Fig. 9). Here, the signal can be fitted by a mono-exponential decay with a time constant *τ* ∼ 5.37 ns. We have performed such experiments on several GQDs. The TR-PL signal is always mono-exponential with relaxation times ranging from 3 to 5.5 ns.

**Fig. 3:**
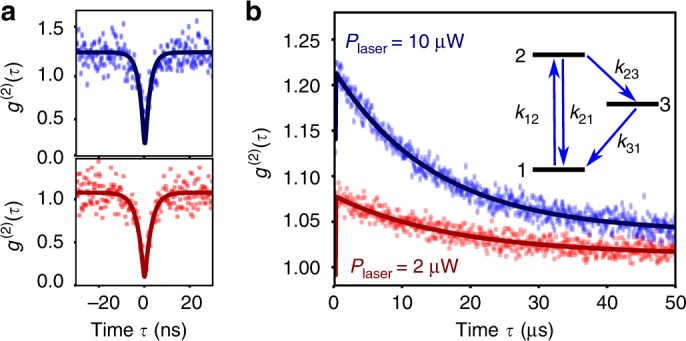
Photons bunching of a single GQD. **a**, **b** *g*^(2)^ functions of a single GQD, for two different excitation powers, 2 μW (red square) and 10 μW (blue diamond). **a** Zoom on short delays; **b** Full timescale intensity correlation. The solid line is a fit to the function $1 - (1 + a)e^{ - \lambda _1|\tau |}$ + $ae^{ - \lambda _2|\tau |}$, convolved by the time response of the detector. The three-level system used as a model is shown

Finally, in order to get more information on the photophysics of GQDS, we studied the *g*^(2)^ function on a longer timescale. In particular, such measurement allows highlighting ISC dynamics between singlet and triplet states^27^. This approach is also supported by calculations on GQDs showing that at least one triplet state is lying few hundreds of meV below the singlet state^28,29^. The *g*^(2)^ functions recorded under different excitation density both at short and long-time delays are shown, respectively, in Fig. 3a, b. At short delays, one observes an acceleration of the dynamics of the *g*^(2)^ function when the pump is increased. Likewise, at longer delays photon bunching (*g*^(2)^ > 1) is observed with a relaxation down to *g*^(2)^ = 1 within tens of microseconds. These data are well fitted assuming a three-level system model, as shown in the inset of Fig. 3b where state 3 stands for the triplet state. It leads to the evaluation of the transition rates: *k*_21_ ∼ 0.28 ns^−1^, *k*_23_ ∼ 0.025 μs^−1^, and *k*_31_ ∼ 0.057 μs^−1^ (see Supplementary Note 1 for the details of the photophysics). The ISC yield *k*_23_/*k*_21_ being small (∼10^−4^) and the triplet lifetime being short (1/*k*_31_ ∼ 18 μs), the effect of the triplet on the emission efficiency is very limited. It can explain both the high brightness of GQDs and the absence of measurable blinking (see also Supplementary Fig. 12). Finally, we also provide an estimation of the absorption cross section of the C_96_ GQD (*σ* ≃ 1.0 × 10^−14^ cm^2^), as well as of the PL quantum efficiency *η*_*Q*_ above 35% (see Supplementary Note 2).

To conclude, the good properties of GQDs: single-photon emission, brightness, and photostability show that they are very promising materials for applications requiring single emitters. More generally, the results reported here demonstrate that the high potential of GQDs revealed by theory is now accessible experimentally. In particular, the observation of single GQD emission paves the way to studies linking their properties to their structure. In addition, our results show that the modification of the properties through the control of the structure is indeed achievable. Therefore, one can now really imagine to perform engineering of other properties such as the spin structure in order to rend it optically detectable and controllable.

## Methods

### Techniques

MALDI-OF spectra were performed with a Perspective Biosystems Voyager DE-STR at the I.C.S.N. (CNRS of Gif-sur-Yvette). ^1^H-NMR spectra were recorded on Bruker AC-300 spectrometer (300 MHz) with CDCl_3_ as reference solvent.

### Materials

Chemicals were purchased from Aldrich and were used as received. The 3,4-bis(4-dodecylphenyl)-2,5-diphenylcyclopentadienone was prepared according to the method described in the literature^30^. Solvents were purchased from Aldrich, VWR or ThermoFisher and were used as received.

### Synthesis

See Supplementary Methods.

### Sample preparation

GQDs powder was dispersed in 1,2,4-trichlorobenzene (TCB) by stirring at least 24 h at room temperature. For single-molecule measurements, a 2 mL diluted GQDs solution (0.001 mg mL^−1^) was mixed with a 2 mL 1,2,4-trichlorobenzene solution (0.08 mg mL^−1^) of polystyrene (PS). Approximately 20 μL of the GQD-PS-TCB solution was spin-coated on an oxygen-plasma-treated glass coverslip for 180 s at 2000 r.p.m. The sample was dried by heating to 90 °C for 1 h on a hot plate. The thickness of the layer has been estimated to 25–50 nm by profilometry.

### Optical measurements

Optical experiments were performed on a home-built micro-PL setup under ambient conditions, as shown in Fig. 1b. The excitation source was a continuous-wave diode laser at 594 nm (Cobolt, Mambo 100) with linear polarization. The excitation laser was focused onto the sample with a high numerical aperture oil-immersion microscope objective (NA = 1.42, Olympus PLAPON 60XO) mounted on a piezoelectric XYZ scanner (Mad City Labs Inc.). The luminescence light was collected by the same objective and filtered from the residual excitation laser using a dichroic mirror (zt 594 RDC, Chroma) and a long-pass filter (FELH0600, Thorlabs). The collected luminescence was then focused on a 50-μm-diameter pinhole and finally directed either into a spectrometer (SP-2358, Princeton Instruments) coupled with a cooled CCD camera (PyLoN:100BR eXcelon, Princeton Instruments) or into two silicon avalanche photodiodes (SPCM-AQR-13, PerkinElmer) mounted in a Hanbury Brown and Twiss (HBT) configuration. Short-time-scale second-order photon correlation measurements were done using a time-correlated single-photon counting module (PicoHarp300, PicoQuant). Long-time-scale second-order photon correlation measurements were done using a wide-range time digitizer (P7887, FastComtec). For time-resolved PL measurements, the sample was excited using a supercontinuum laser (Fianium) tuned at 580 nm by an acousto-optic tunable filter systems with a 6 ps pulse width and a 60 MHz repetition rate.

## Electronic supplementary material

Supplementary Information

Peer Review file

## Data Availability

The data that support the findings of this study are available from the corresponding author upon reasonable request.
