# Supplementary material for: Single photon emission from graphene quantum dots at room temperature
Source: Nat Commun. 2018 Aug 27;9:3470. doi: 10.1038/s41467-018-05888-w (PMC6110725; doi:10.1038/s41467-018-05888-w)
Supplement: Supplementary file 1 — Supplementary Information [file 41467_2018_5888_MOESM1_ESM.pdf]

Supplementary information: Single photon emission from graphene quantum dots at room temperature

S. Zhao,[1] J. Lavie,[2] L. Rondin,[1] L. Orcin-Chaix,[1,2] C. Diederichs,[3] Ph. Roussignol,[3] Y. Chassagneux,[3] C. Voisin,[3] K. Müllen,[4] A. Narita,[4] S. Campidelli,[2] and J.S. Lauret[1,*]

[1]*Laboratoire Aimé Cotton, CNRS, Univ. Paris-Sud, ENS Cachan, Université Paris Saclay bat 505 campus d'Orsay, 91405 Orsay cedex France*
[2]*LICSEN, NIMBE, CEA, CNRS, Université Paris-Saclay*
[3] *Laboratoire Pierre Aigrain, Département de physique de l'ENS, École normale supérieure, PSL University, Université Paris Diderot, Sorbonne Paris Cité, Sorbonne Université, CNRS, 75005 Paris, France*
[4]*Max Planck Institute for Polymer Research, Ackermannweg 10, 55128 Mainz, Germany*

Supplementary Note 1: Modeling the photophysics

Three-level system. We describe the GQD response within the framework of a three-level system: ground state (level 1), excited state (level 2) and metastable state (level 3) as shown in Fig.3 of the main text. The transition rate from level n to level m is given by k_{nm} (with $n, m = 1, 2, 3$). Assuming that k_{23} and k_{31} are very small compared to k_{21}, the normalized second-order auto-correlation function $g^{(2)}(t)$ can be expressed as [1]:

$$g^{(2)}(t) = 1 - (1 + a)e^{-\lambda_1 t} + ae^{-\lambda_2 t} \quad (1)$$

with following expressions for the parameters a, λ_1, and λ_2:

$$\lambda_1 = k_{12} + k_{21} \quad (2)$$
$$\lambda_2 = k_{31} + k_{23}k_{12}/(k_{12} + k_{21}) \quad (3)$$
$$a = k_{23}k_{12}/\left[k_{31}(k_{12} + k_{21})\right] \quad (4)$$

Note that the usual "*start-stop*"setup that serves to measure the $g^{(2)}$ function is only valid on timescale smaller than R^{-1}, where R is the counting rate [2]. In order to capture the full dynamics of the GQD photophysics, and especially the long bunching characteristics time (${\lambda_2}^{-1} \sim \mu$s), we measure $J(t)$ which is the histogram of photons detected at time t provided that a photon is detected at time $t = 0$. To this end, a photon detection event on detector D_1 was used to trigger the acquisition of a PL time trace on detector D_2 using a wide-range time digitizer (P7887, FastComtec). After N repetitions of the measurement, the resulting histogram $J(t)$ is directly linked to the $g^{(2)}(t)$ function through [3, 4]:

$$g^{(2)}(t) = \frac{J(t)}{NwR_2} \quad (5)$$

where w is the time bin width and R_2 is the photon count rate on detector D_2.

Supplementary Fig. 11A represents a typical recorded $g^{(2)}(t)$ function over 50 μs with an excitation power of 10 μW. We observe that the $g^{(2)}(0)$ at zero time delay has a non-negligible residual value for excitation power larger than a few μW. This deviation from $g^{(2)}(0) = 0$ is due to the shortening of the antibunching characteristic time ${\lambda_1}^{-1}$ with the incident power bringing it closer to the instrument response [5]. Using 6 ps supercontinuum laser pulses, the instrument response function (IRF) was independently measured as a 0.9 ns FWHM Gaussian function (Supplementary Fig. 11B). The measured $g^{(2)}$ function is then well fitted by the convolution of the IRF with Eq. 1.

To gain insights into the transition rates, we measured the $g^{(2)}$ function of a GQD at different excitation powers. By fitting the $g^{(2)}$ functions, we obtain the values of λ_1, λ_2 and a. We plot these values as a function of excitation power (Supplementary Fig. 11C, D and E, respectively). From these plots, we can deduce all of the transition rates.

The population rate of excited state k_{12} is linked to the excitation power P by the relation:

$$k_{12} = \sigma P/h\nu \quad (6)$$

where σ is the absorption cross-section and ν is the excitation frequency. Combining this relation with Eq .2, we fit the λ_1 values with a linear function (see Supplementary Fig. 11C). From this fit, we obtained the absorption cross-section $\sigma \simeq 1.0 \times 10^{-14}$ cm^2. By extrapolating the linear fit to zero excitation power, we also obtain the relaxation rate of the excited state $k_{21} = 0.28 \pm 0.02$ ns^{-1}. The lifetime of excited state ${k_{21}}^{-1}$ is thus calculated to be 3.55 $\pm$ 0.24 ns. This value is compatible with our time-resolved PL measurements leading to life times ranging from 3 to 6 ns depending on the GQD.

Using Eq. 3 and 4, the relaxation rate of metastable state k_{31} is given by

$$k_{31} = \frac{\lambda_2}{1+a} = 0.053 \pm 0.001 \ \mu\text{s}^{-1} \quad (7)$$

We then fit the λ_2 by combining Eq. 3 and 6. Treating k_{23} as a constant, we obtain $k_{23} = 0.025 \pm 0.005$ μs^{-1}.

Supplementary Note 2: Estimation of the quantum yield

We now estimate the quantum yield of GQD. Using the transition rates, the detected fluorescence rate can be expressed as [5]

$$R = \eta_{det}\eta_Q \frac{k_{21}}{(k_{21}/k_{12}+k_{23}/k_{31}+1)} \quad (8)$$

where R is the total count rate, η_{det} is the overall detection efficiency and η_Q is the fluorescence quantum yield. By fitting the fluorescence saturation curve, together with the deduced transition rates, we obtain $\eta_{det} \times \eta_Q \approx 0.05$. In Tab. 1 we list the transmission or collection efficiency of each optical component in the detection path, leading to an overall collection efficiency expected to be lower than 15%. Therefore, a lower bound of the fluorescence quantum yield is $\sim 35\%$.

objective lens	mirrors	dichroic mirror	beamsplitter
30%	92%	98%	95%
lenses	optical filters	APD	η_{det}
97%	88%	70%	$\sim$ 15%

Supplementary Note 3: Comparison the count rate of GQD with other quantum emitters
In order to compare GQDs with other quantum emitters, we first performed experiments on a single NV center in a 111-diamond on the same experimental setup as the one used for GQDs. A $R_{\text{sat}} \approx 0.3$ Mcounts/s is measured (see Supplementary Fig. 6). Since L.J. Martìnez et al have compared on the same setup the count rate at saturation of N-V centers and single defects in h-BN [4], it allows us to compare GQDs with defects in h-BN. They measure $R_{\text{sat}} \approx 4$ Mcounts/s for single defects in h-BN. Therefore, we can conclude that GQDs are, at least, as bright as these new quantum emitters that are considered as one of the brightest single photon source.

Supplementary Note 4: Fraction of off-time.
To study the fraction of off-time as a function of pump power, we record individual photon detection events with their arrival time in time-tagged time-resolved (TTTR) mode of a PicoHarp 300. We then obtain an histogram of the inter-event waiting times. For a Poissonian source, the inter-event waiting time distribution is a single exponential with decay time τ equal to the inverse of the count rate. If the source exhibits blinking with off-time on a time scale $\tau_{\text{blink}} > \tau$, the waiting time distribution should have an additional decay with characteristic time determined by τ_{blink}. At low excitation power (200 nW), the waiting time distribution of GQDs can be fitted with a single exponential with decay time τ of 25 μs (see Supplementary Fig.12A), in good agreement with the detection count rate of 40 kcounts/s. It implies that there is no blinking on a time scale longer than 25 μs. At high excitation power, the histogram exhibits a fast decay, directly linked to the detection count rate, combined with an additional slower decay of 10 μs (see Supplementary Fig.12B). The fast decay is about 4 order of magnitude stronger than the slower one, which is closed to the value of the intersystem crossing yield $k_{23}/k_{21} \sim 10^{-4}$ deduced from the photophysic analysis (see above). Moreover, the value of the slower decay (10 μs) is in good agreement with the triplet lifetime $1/k_{31} \sim 18$ μs. We thus conclude that the intensity fluctuations only originate from the passage to the triplet state. Due to the short triplet lifetime and low intersystem crossing yield, the effect on the emission efficiency is very limited.

Supplementary Note 5: Bleaching of GQDs.
We looked on few cases whether the GQDs emission is irreversibly quenched or if they are able to re-emit light after a certain time. Most of the time, the GQD remains dark. We found one situation where the GQD re-emit light after its bleaching with a slight modification of its emission spectrum.

Supplementary Methods: Synthesis of GQDs
Dendrimer **3**. 3,4-bis(4-dodecylphenyl)-2,5-diphenylcyclopentadienone (450 mg, 0.62 mmol) and the 1,3,5-triethynylbenzene (25 mg, 0.17 mmol) were introduced in a dry Schlenck flask. After two vacuum-argon cycles, o-xylene (2 mL) were added and the reaction was left at 180 °C overnight. The suspension was diluted in dichloromethane (2 mL) and the solution was precipitated in cold ethanol (200 mL). After filtration on PTFE membrane (0.2 μm), the product was redispersed in hot methanol to solublize the excess of cyclopentadienone, filtered on PTFE membrane and washed with methanol. Finally, 220 mg of yellowish powder were obtained (57% yield). ^{1}H NMR (300MHz, $CDCl_3$): 7.10-6.60 (60H, m, Ar-H); 2.37 (12H, q, J=5.6 Hz, $6CH_2$(Ar)dodecyl); 1.27 (120H, s, $60CH_2$-dodecyl); 0.90 (18H, t, J=5.6 Hz, $6CH_3$-dodecyl). MS (MALDI-TOF): m/z calcd for $C_{168}H_{210}$: 2227.64; found: 2227.60 (M^+).

C_{96} GQD. Dendrimer **3** (50 mg, 22.4 μmol) was dispersed in non-stabilized dichloromethane (40 mL) in a two-necked round bottom flask of 100 mL. Separetly, $FeCl_3$ (458 mg, 2.8 mmol) was dissolved in anhydrous nitromethane in a gloves box (2 mL) and then added to the solution of dendrimer. The solution was left 18 hours under argon coming from a two necked round bottom flask filled with dichloromethane in which argon was bubbling. The evolution of the reaction was followed with MALDI-TOF mass spectroscopmetry after quenching of an aliquot with methanol. When the reaction was finished, the solution was quenched with methanol (40 mL) and then filtered on PTFE membrane (0.2 μm). 46 mg of a black powder were obtained as the pure product (95% yield). MS (MALDI-TOF): m/z calcd for $C_{168}H_{174}$: 2191.36; found: 2191.87 (M^+).

Dendrimer **5**. The tetraphenylcyclopentadienone (240 mg, 0.63 mmol) and the 1,3,5-triethynylbenzene (26 mg, 0.17 mmol) and o-xylene (2mL) were introduced in a dry Schlenck flask and heated at 180 °C for a night.

The viscous violet product was diluted with 1mL of dichloromethane and added dropwise to cold ethanol (200mL). The violet product was filtered on PTFE (0.2μm) and dried under vacuum. The product was first purified with column chromatography with toluene to get rid of the Ph_2O. The product was redispersed in hot methanol to solublize the excess of cyclopentadienone, filtered on PTFE membrane and washed with methanol. Finally, 306.4 mg of white powder were obtained (40% yield). MS (MALDI-TOF) m/z: calcd for $C_{96}H_{66}$: 1219.58 found: 1219.54

GQD 6. The dendrimer $C_{96}H_{66}$ (268 mg, 0.22 mmol) was dispersed in 120mL of non-stabilized dichloromethane in a two-necked round bottom flask of 250 mL. Separetly, $FeCl_3$ (4.49 g, 27.6 mmol) was added to 5mL of anhydrous nitromethane in a glovebox and then added to the solution of dendrimer. The solution was left 18 hours under Argon coming from a two necked round bottom flask filled with Dichloromethane in which argon was bubbling. The evolution of the reaction was followed with MALDI-TOF mass spectroscopy after quenching of the sample with methanol. The solution was quenched with methanol (about 80mL) and then filtered on PTFE and washed with cold methanol. Finally, 247.7 mg of black powder were obtained (95% yield). MS (MALDI-TOF) m/z: calcd for $C_{96}H_{30}$: 1182.23, found: 1182.30.

C_{96}Cl GQDs. GQD 6 (29 mg, 0.025 mmol) and $AlCl_3$ (13 mg, 0.098 mmol) were introduced in a dry two necked round bottom flask (100mL), and dissolved in 20 mL CCl_4. After 20 min of Argon bubbling, ICl (0.74 mL, 14.7 mmol) were added and the reaction was left for 48h at 80 °C. The reaction was quenched with ethanol and ICl and CCl_4 were evaporated at about 60 °C with a liquid nitrogen trap. The solid obtained after evaporation of the solvents was then washed with ethanol and purified by column chromatography with chloroform as eluent. Finally, 37.2mg of violet powder were obtained (72% yield). MS (MALDI-TOF) m/z: calcd for $C_{96}H_3Cl_{27}$:2113.28, found: 2113.72.

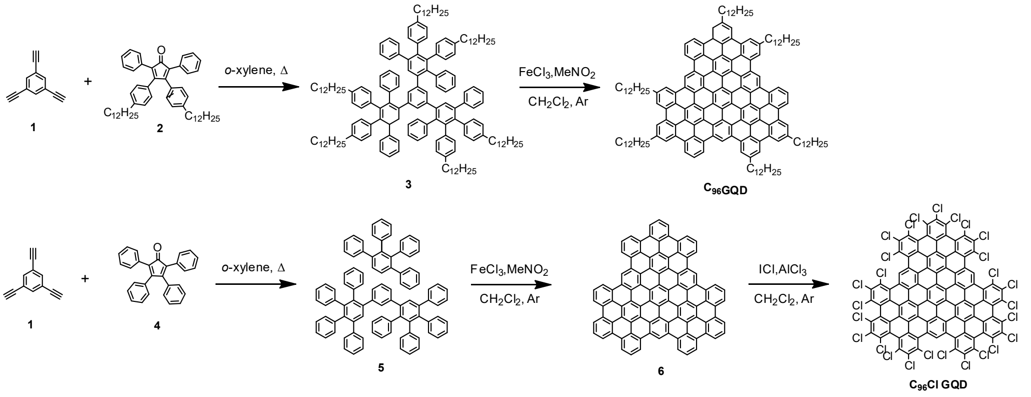

Supplementary Fig. 1: Reaction routes to the production of C_{96} and C_{96}Cl GQDs.

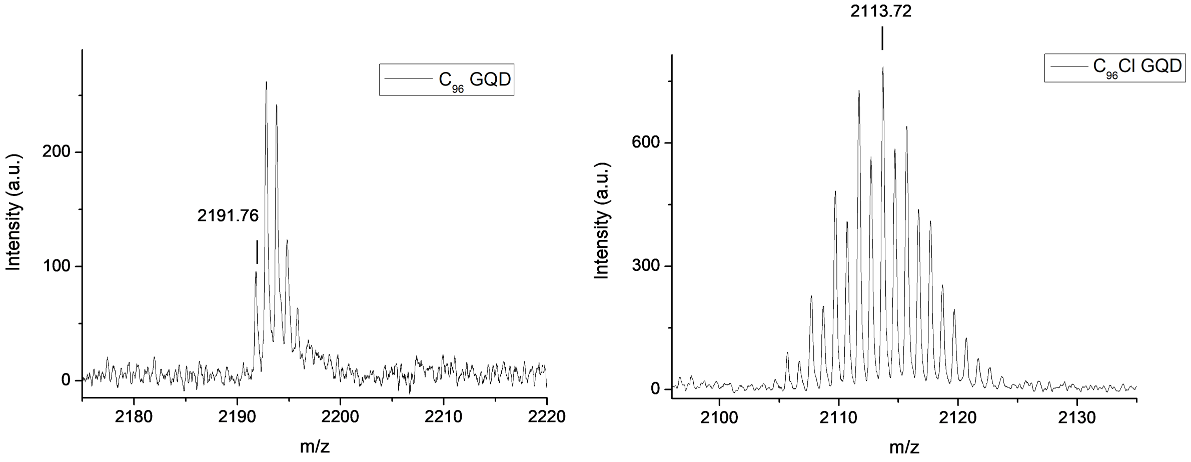

Supplementary Fig. 2: **MALDI-TOF mass spectra** of C_{96} and C_{96}Cl GQDs. The spectra do not show the presence of starting or incompletely oxidized materials.

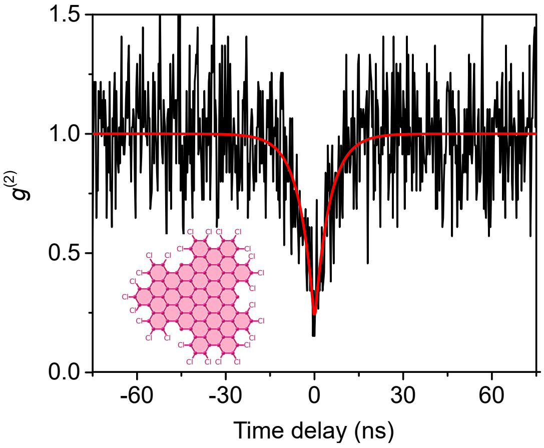

Supplementary Fig. 3: $g^{(2)}$ curve of the $C_{96}Cl$ GQD whose spectrum is displayed on Fig.1c. Here the edge chlorination leads to an almost 100 nm redshift of the main PL line while maintaining a single photon emission. Inset: Schematic chemical structure of the $C_{96}Cl$ GQDs.

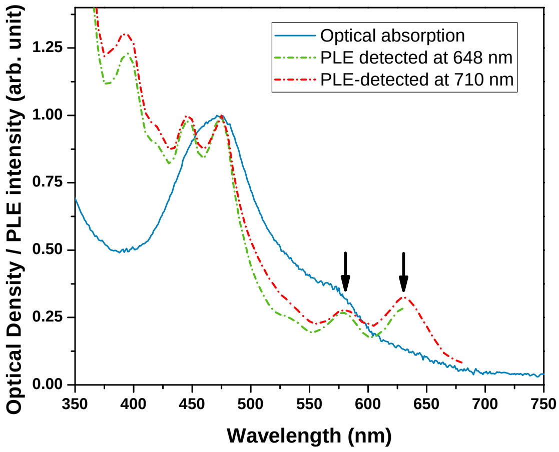

Supplementary Fig. 4: Optical absorption spectrum of C_{96} GQDs in solution in trichlorobenzene (blue) and PLE spectra (green and red). The PLE was perfomed using a supercontinuum laser (Fianium) filtered by a monochromator as excitation source. The PL signal is then analyzed in spectrometer coupled to a CCD. The absorption spectrum is composed of a main line at ∼475 nm and of some shoulder on the low energy tail. Likewise, both PLE spectra recorded on the two main PL lines follow the trend of the absorption spectrum. Moreover, PLE lines are much more structured than the absorption spectrum. This may be an indication that some aggregates still remain in the suspension. Their PL quantum yield would be much lower than the one of monomers, explaining why the PL spectrum is dominated by the monomers.

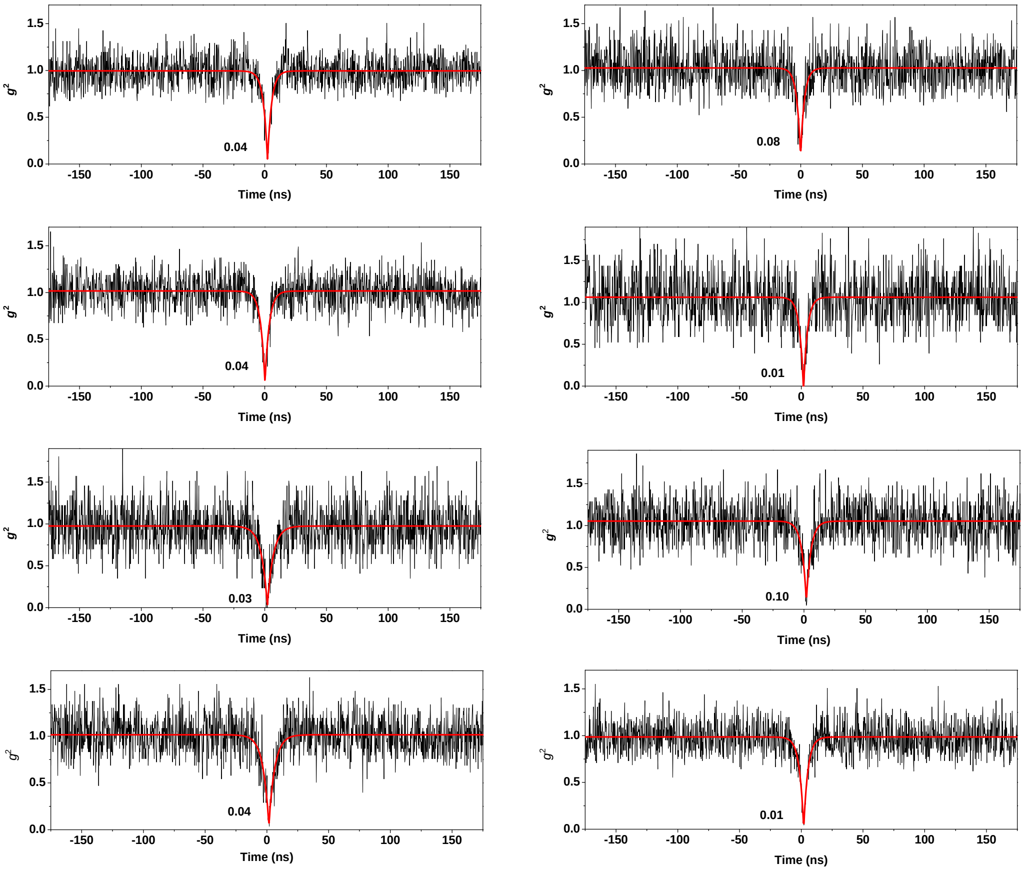

Supplementary Fig. 5: Collection of $g^{(2)}(\tau)$ traces on different GQDs recorded at moderate excitation power ($\approx$ 200 nW).

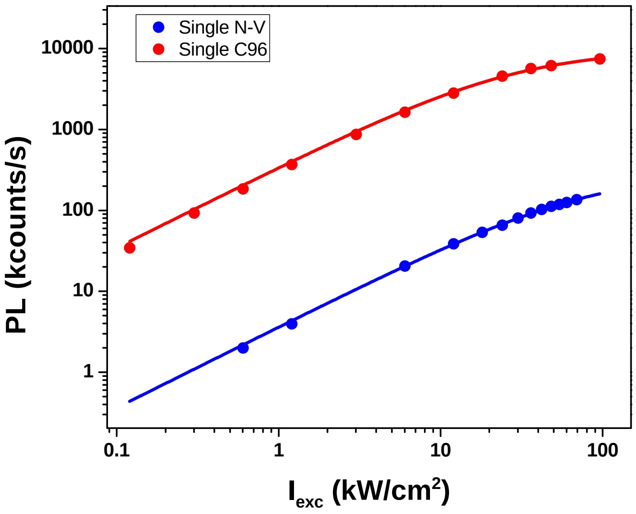

Supplementary Fig. 6: Saturation curves of a GQD (red dots) and a single NV center in diamond (blue dots). Both were fitted with the saturation functions described in the main text.

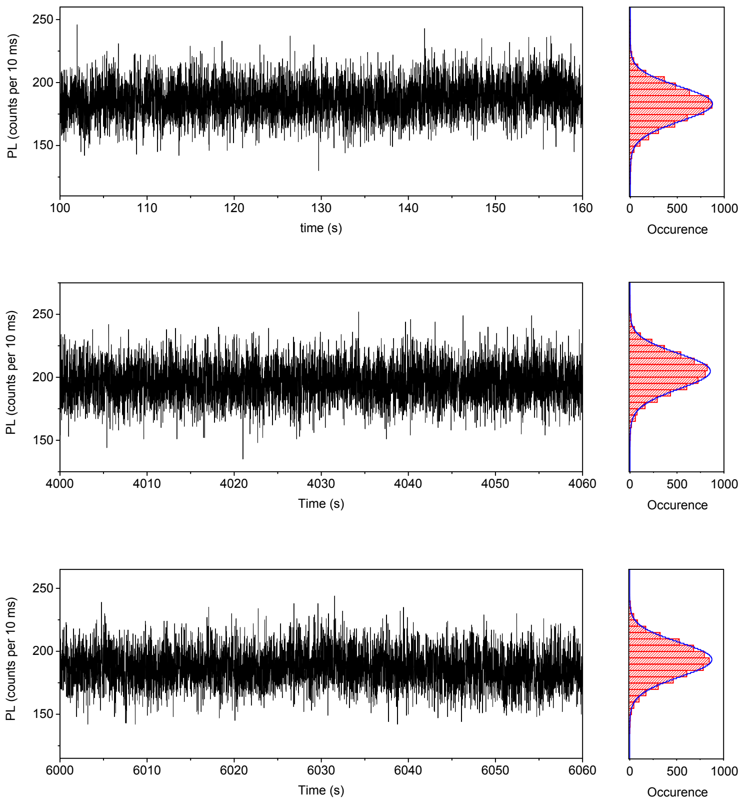

Supplementary Fig. 7: **Intensity histograms** PL time traces (the same as shown in the zooms of Fig. 2d of main text) and corresponding count rate histograms. The histograms were fitted by normal functions (blue curves)

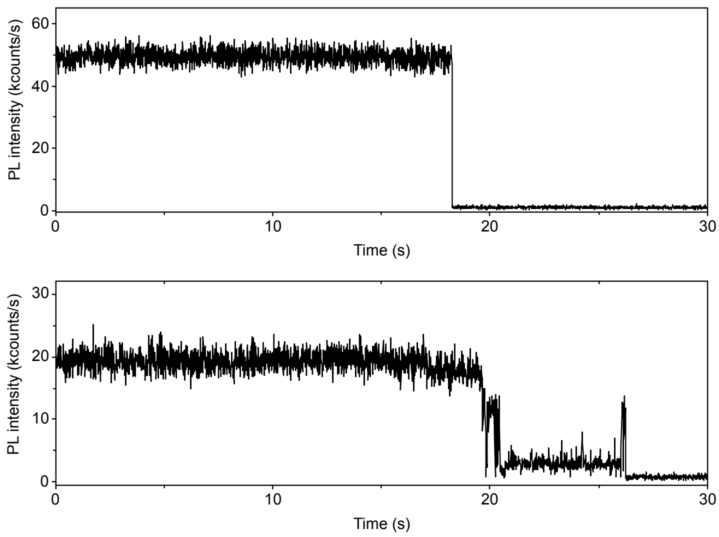

Supplementary Fig. 8: **PL time traces** recorded from different GQDs around the time of bleaching. Bin size: 10 ms.

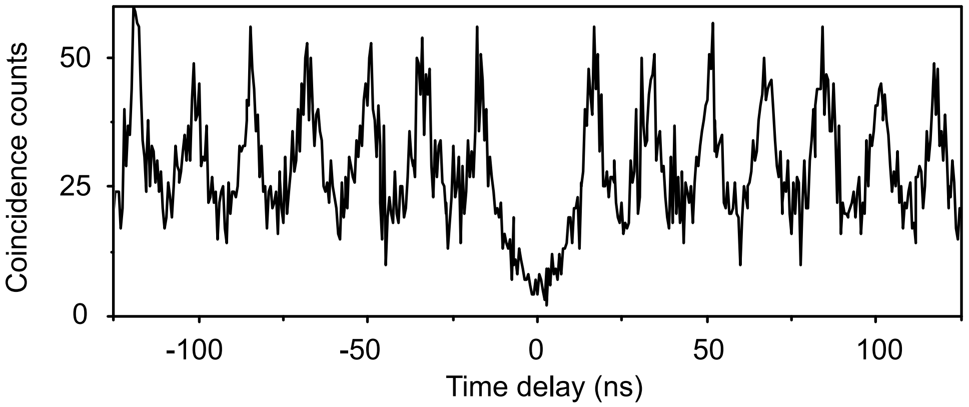

Supplementary Fig. 9: $\mathbf{g}^{(2)}(\tau)$ **trace under pulsed excitation** (580 nm) on the same GQD as the one corresponding to the PL decay curve of Fig. 2b of the main text.

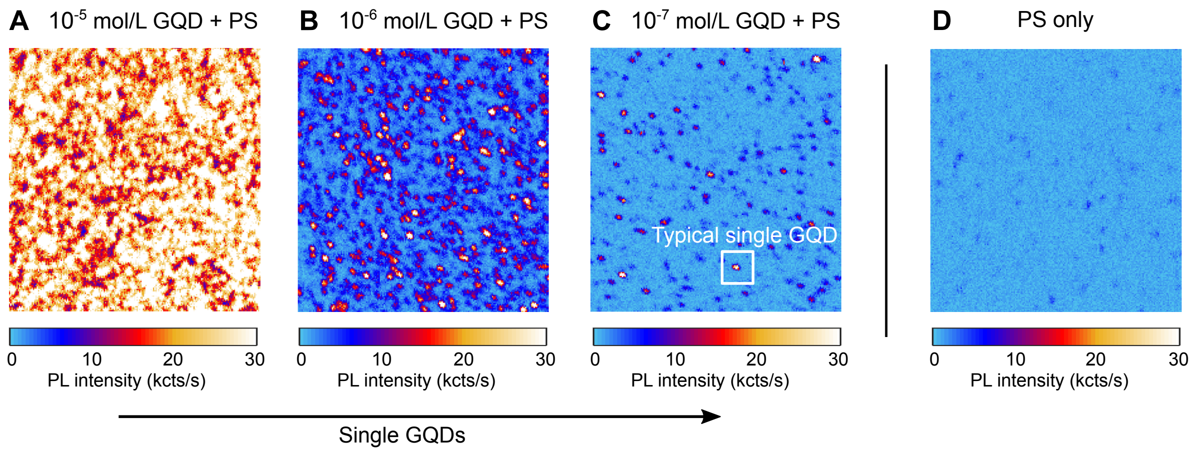

Supplementary Fig. 10: **PL maps of GQDs** in a polystyrene matrix spin-coated from solutions at different concentrations of C_{96} GQD: **A** 10^{-5}mol.L^{-1}; **B** 10^{-6}mol.L^{-1}; **C** 10^{-7}mol.L^{-1}. **D** PL map of polystyrene film without GQDs. Map size: 20×20 μm^2. When a highly concentrated solution is used to make the thin film, luminescence is observed everywhere on the sample. When the concentration decreases, the spatial distribution of PL is more and more localized. It ensures that the bright luminescence spots do arise from GQDs. Moreover, the **D** panel shows a map of a polystyrene film without GQDs. It shows very weak PL signal in comparison with the GQD samples. The localized weak PL spots that are still observed bleach quasi instantaneously, in strong contrast with the GQDs spots that are stable for hours.

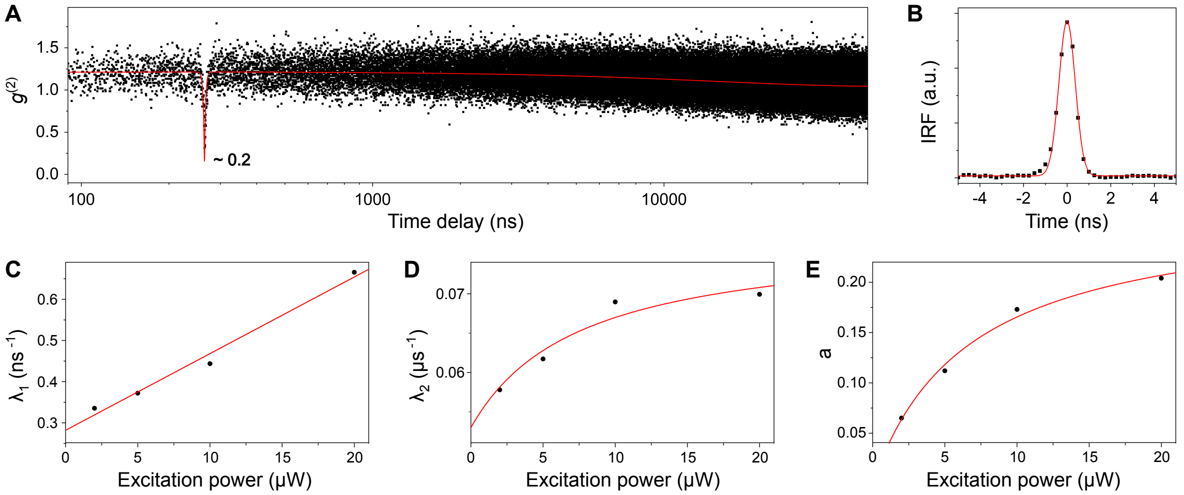

Supplementary Fig. 11: **Photophysics of a single GQD**. **(A)** Long-time-scale $g^{(2)}$ function recorded with an excitation power of 10 μW. Bin width: 250 ps. A time delay is introduced for log-scale display. The red line is a fit by the convolution of Eq.1 and the measured instrument response function (IRF). **(B)** Impulse Response Function of the detector measured using 6-ps laser pulses. The Gaussian fit (red line) gives a FWHM of 0.9 ns. **(C)-(E),** Intensity dependence of the fit parameters λ_1 (c), λ_2 (d) and a (e) of $g^{(2)}$. The red lines are fits according to the three-level model.

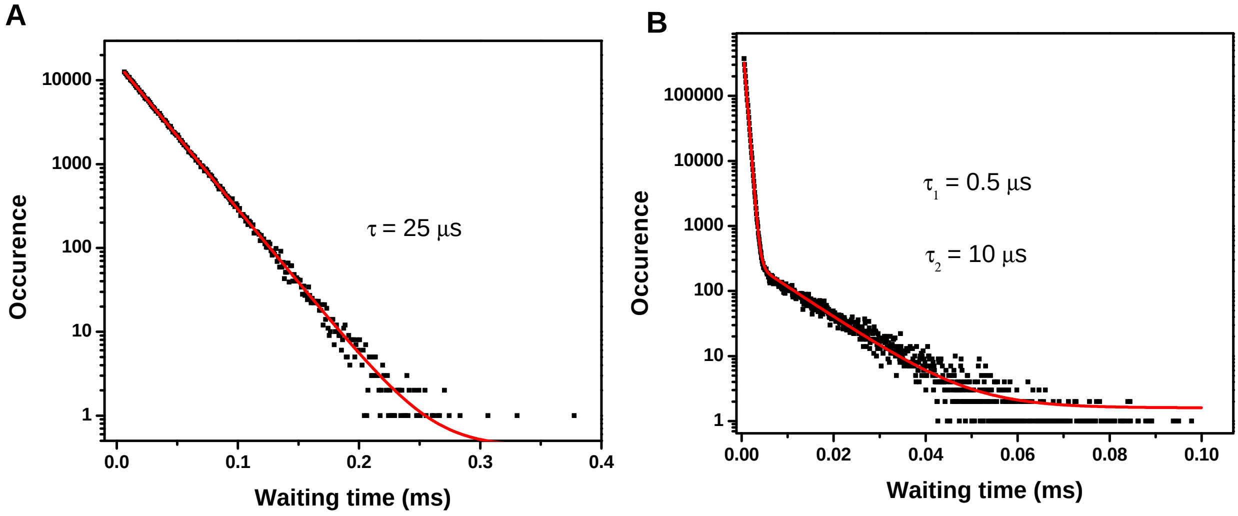

Supplementary Fig. 12: **Fraction of off-states**. Histograms of the inter-event waiting times **(A)** at low excitation power. Fit with a monoexponential decay (red curve); **(B)** At high excitation power. Fit with a bi-exponential decay (red curve)

Supplementary References

---

* Electronic address: lauret@ens-paris-saclay.fr

[1] L. Novotny, B. Hecht, *Principles of Nano-Optics* (Cambridge University Press, 2012), second edn.
[2] Reynaud, Serge, *Ann. Phys.* **8**, 315 (1983).
[3] C.-H. Huang, Y.-H. Wen, Y.-W. Liu, *Opt. Express* **24**, 4278 (2016).
[4] L. J. Martínez, *et al.*, *Phys. Rev. B* **94**, 121405 (2016).
[5] E. Wu, *et al.*, *Opt. Express* **14**, 1296 (2006).

****
