# Supplementary material for: Single photon emission from graphene quantum dots at room temperature
Source: Nat Commun. 2018 Aug 27;9:3470. doi: 10.1038/s41467-018-05888-w (PMC6110725; doi:10.1038/s41467-018-05888-w)
Supplement: Supplementary file 2 — Peer Review file [file 41467_2018_5888_MOESM2_ESM.pdf]

Reviewer#2:
The authors have made many corrections and improvements that make the work suitable for publication. In its final form, the manuscript makes it clear that the so-called graphene quantum dot behaves more as an aromatic molecule. To this end, reference to stable emission from such molecules at room temperature should be made (examples: Pfab, et al, Chem. Phys. Lett. 387, 490 (2004) and C. Toninelli, et al, Opt. Express 18, 6577 (2010) should be made).

We thank reviewer#2 for his/her comments. We add the two references in the main text.

Reviewer#3:
The authors have adequately addressed my questions and comments. I recommend publication in Nature Communications.

We thank reviewer#3 for his/her comments.

Since all reviewers recommend our paper for publication, we hope that you will able to accept this final version of our paper for publication in *Nature Communications*.

Best regards,
